# Intradural prepontine chordoma in an 11-year-old boy. A case report

**DOI:** 10.1007/s00381-015-2818-z

**Published:** 2015-07-28

**Authors:** R. Saman Vinke, Elise Charlotte Lamers, Benno Kusters, Erik J. van Lindert

**Affiliations:** Department of Neurosurgery, Radboud University Medical Center, P.O. Box 9101, 6500 HB Nijmegen, The Netherlands; Department of Neurosurgery, St. Elisabeth Hospital, Tilburg, The Netherlands; Department of Pathology, Radboud University Medical Center, Nijmegen, The Netherlands

**Keywords:** Chordoma, Intradural, Prepontine, Paediatric

## Abstract

**Case report:**

The authors report a case of an 11-year-old boy that presented with headache and vomiting that was present for several months. CT and MR imaging revealed a large prepontine mass and an obstructive hydrocephalus. A ventriculoperitoneal shunt was inserted, and in a second operation, a radiologically proven total resection was performed, using a left frontotemporal transsylvian approach. The tumour showed no involvement of the dura or clivus. Histological examination showed the characteristics of a chordoma. No further adjuvant treatment was given. The patient remained disease or tumour free after a 6-year follow-up.

**Discussion:**

Intradural chordomas are extremely rare tumours that originate from notochordal remnants. Only three other cases have been reported in the paediatric population. Ecchordosis physaliphora (EP) is an ectopic notochordal remnant that has a similar biological behaviour and is difficult to distinguish from intradural chordomas. They might exist in a continuum from benign notochordal tumour to malignant chordoma. A surgical resection without adjuvant radiation therapy is suggested to be the treatment of choice in the paediatric population.

**Conclusion:**

The authors describe a rare case of an intradural prepontine chordoma in an 11-year-old boy that stayed disease free after a 6-year follow-up.

## Introduction

Chordomas are rare, slow-growing tumours accounting for 1–4 % of all primary malignant bone tumours [11]. They usually occur in adults with a peak incidence between 50 and 60 years of age [16]. The incidence in children and adolescents is less than 5 % of the total incidence [4]. Chordomas are thought to arise from notochordal remnants throughout the axial skeleton and are most commonly located in the skull base, mobile spine and sacrum at an almost equal distribution [16]. Although intradural localization of chordomas is rare, several cases have been reported in the literature. However, most of the reported cases have occurred in adults and only three cases of a pure intradural chordoma in a paediatric patient have been reported [5, 9, 27]. In this report, we present a case of an intradural prepontine chordoma in an 11-year-old boy with a 6-year follow-up and an update of the literature.

## Case report

**Fig. 1:**
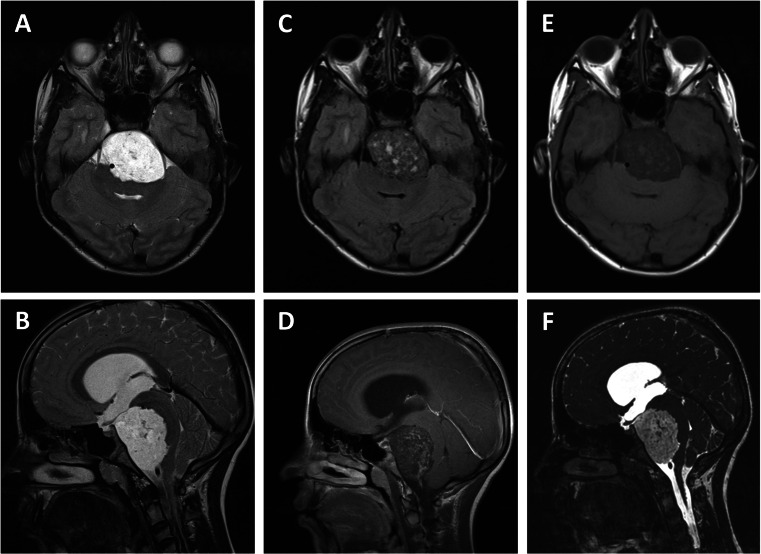
Preoperative MR images. **a**, **b** Axial and parasagittal T2-weighted MRI scans showing an inhomogeneous hyperintense prepontine lesion with a mass effect on the brainstem. **c**, **d** Axial and parasagittal T1-weighted MRI scan after administration of gadolinium contrast show inhomogeneous enhancement. **e** Axial T1-weighted MRI scan shows an inhomogeneous hypointense prepontine lesion. **f** Parasagittal constructive interference steady state (CISS) MRI scan shows an inhomogeneous hyperintense prepontine lesion

An 11-year-old boy presented with a history of headaches and vomiting that had been present for several months. Physical examination showed papilledema without the presence of any other neurological deficits. Magnetic resonance imaging (MRI) revealed a large prepontine mass with dorsal displacement of the brainstem and a secondary obstructive hydrocephalus due to compression of the aqueduct. The lesion had an inhomogeneous hypointense aspect on the T1-weighted image (T1WI) and an inhomogeneous hyperintense aspect on the T2-weighted image (T2WI). After administration of IV gadolinium, there was some inhomogeneous enhancement (Fig. 1). Computed tomography (CT) imaging showed no bone involvement.

### Operation

During the first operation, a ventriculoperitoneal shunt was inserted into the right lateral ventricle to treat the hydrocephalus. A careful study of the MRI suggested that this infra- and supratentorially located tumour might be resected through a single approach. A left frontotemporal transsylvian approach was performed to gain access to the tumour. The tumour had well-defined margins and was entirely located in the intradural plane. There were no attachments to the cranial nerves or brainstem. A macroscopic complete resection was performed.

### Postoperative course

**Fig. 2:**
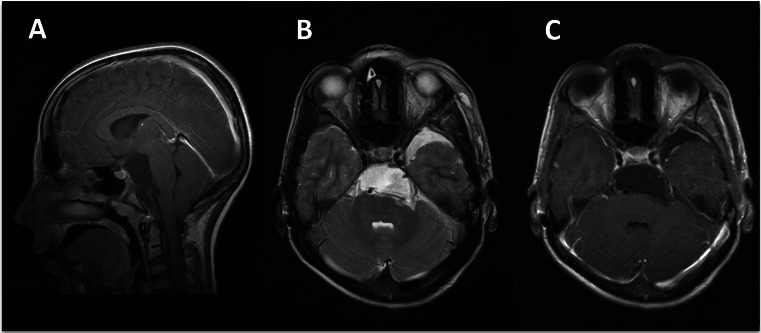
Postoperative MR images. A complete tumour removal is shown on the **a** parasagittal T1-weighted sequence, **b** axial T2-weighted sequence and **c** axial T1-weighted sequence after administration of gadolinium contrast

Postoperatively, the patient had developed a left oculomotor nerve palsy, which completely recovered within the next 4 weeks. The postoperative MRI showed a complete removal of the tumour (Fig. 2). After careful consideration by a multidisciplinary team, we decided that there was no indication for postoperative radiation therapy. At follow-up one and a half years later, the patient was found to have remained asymptomatic. There were no signs of tumour recurrence on the MRI scan. At a follow-up of more than 6 years after treatment, there were still no signs of tumour recurrence on the MRI scan.

### Histopathological and immunohistochemical findings

**Fig. 3:**
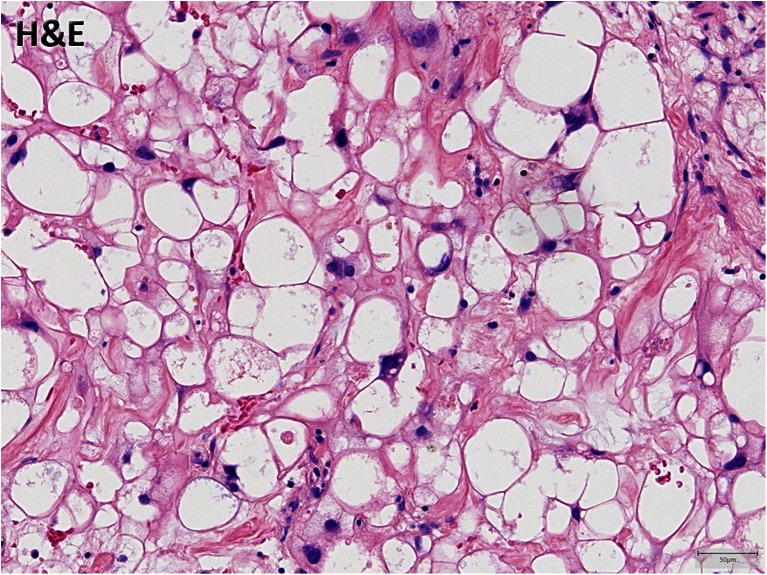
Histopathological imaging. The lesion shows vacuolated tumour cells with a pale cytoplasm and moderate nuclear polymorphism in a chondromyxoid matrix. These findings are a characteristic of chordoma

Histological examination showed a slightly lobulated tumour consisting of a chondromyxoid matrix. The tumour cells showed a vacuolated and pale cytoplasm. Moderate nuclear polymorphism was observed but no obvious mitotic activity (Fig. 3). Some calcifications were seen. The tumour cells stained positive for pan-keratin, S-100 and epithelial membrane antigen (EMA). These findings suggest a histopathological diagnosis of chordoma.

## Discussion

Chordomas are rare primary bone tumours. Despite their low-grade histological characteristics, chordomas often show a malignant disease course [2, 21, 23]. Symptoms usually develop gradually, related to the relatively slow-growing tumour compressing the surrounding structures. However, chordomas can present with acute and progressive clinical symptoms due to intratumoral haemorrhage as well [22]. The extent of initial surgical resection has been shown to be the most important predictor of local tumour recurrence and long-term survival [6, 10, 14, 23].

Chordomas are most commonly located in the extradural space, with possible transdural progression. Purely intradurally localised chordomas are extremely rare with an incidence of 3.8 % of all chordoma cases. Nevertheless, several cases and even small case series have been reported in the literature [3, 20, 24]. To our knowledge, only three other cases of intradural chordoma have been reported in the paediatric population (Table 1).

**Table 1:** Reported cases of intradural chordomas in the paediatric population

Reference	Age (years)	Sex	Location	Resection	RTx	Follow-up	Recurrence
Dow et al. (2003) [9]	9	F	Right cerebellar hemisphere	Complete	No	14 months	No
Chang et al. (2008) [5]	9	M	Right prepontine cistern	Complete	No	12 months	No
Zhang et al. (2010) [27]	8	ND	ND	ND	ND	ND	ND
This case	11	M	Prepontine	Complete	No	6 years	No

*RTx* radiotherapy, *ND* no data available

Intradural chordomas tend to show different biological behaviour compared to classic chordomas. In contrast to classic chordomas, purely intradural chordomas seem to have well-defined tumour margins, do not show bone invasion and are less adhesive to the surrounding structures all of which improves the resectability [20]. This is likely to be a reason for the lower recurrence rate compared to typical chordomas. In the literature, several cases of intradural chordomas are reported in children and adults (for review, see Dow et al. [9] and Masui et al. [15]). None of the reported cases showed tumour recurrence after surgery, even in cases of subtotal resection. In contrast, typical chordomas often show recurrence, metastatic features and progression to death despite adequate surgical resection, which is sometimes followed by adjuvant radiation therapy or chemotherapy [12]. However, AlOtaibi et al. [1] suggest in a recent systematic review that in the adult population, intradural chordomas show a comparable biological behaviour to typical cranial base chordomas.

Chordomas are thought to originate from remnants of the embryological notochord throughout the axial skeleton. Whereas typical chordomas are localised extradurally with possible transdural progression, this pathophysiologic mechanism does not explain a purely intradural localisation of chordomas.

Ecchordosis physaliphora (EP) are ectopic notochordal remnants that are usually asymptomatic. An incidence of 1.5–2 % in autopsy studies and approximately 1.7 % in MRI studies has been found [8, 17, 19]. EPs are typically found in the prepontine cistern, attached to the dorsal clivus with a small pedicle [13, 25]. Other distinctive features are that EPs generally occur in younger people compared to intradural chordomas and that EPs show no enhancement after gadolinium administration on MR imaging, whereas intradural chordomas show a variable enhancement [7, 17]. Although differentiating between symptomatic EPs and intradural chordomas based on histology is difficult and debated, Yamaguchi et al. [26] suggested some histological differentiating features. Symptomatic EPs and intradural chordomas tend to show a similar benign behaviour with a very low risk of tumour recurrence after surgical resection [7]. Chang et al. [5] suggest that EPs and intradural chordomas exist in a continuum from benign hamartomas to aggressive typical chordomas.

Treatment of a classic chordoma involves extensive surgery, sometimes combined with adjuvant radiation therapy [23]. There is not enough data available to postulate an evidence-based treatment for intradural chordomas in the paediatric population. One should consider that all of the reported cases (Table 1) were treated with a complete surgical resection without any adjuvant treatment, and none of these tumours did reoccur. Even cases in adults treated with a subtotal resection without adjuvant therapy did not show tumour recurrence after a 5-year follow-up [18]. Therefore, the role of adjuvant radiation therapy for intradural chordomas is debated. Considering the potential deleterious effect on the paediatric brain, radiation therapy should be avoided in the paediatric population. The authors postulate that a complete surgical resection without adjuvant radiation therapy should be the treatment of choice.

## Conclusion

Although extremely rare, an intradural chordoma should be considered in the differential diagnosis of intradural prepontine lesions. The authors postulate that a complete surgical resection without adjuvant radiation therapy is the treatment of choice and that adjuvant radiation therapy should be avoided in the paediatric population. Additional studies are required to improve our knowledge about intradural chordomas and their treatment.
